# Intimal Arteritis and Microvascular Inflammation Are Associated With Inferior Kidney Graft Outcome, Regardless of Donor-Specific Antibodies

**DOI:** 10.3389/fmed.2021.781206

**Published:** 2021-12-08

**Authors:** Marek Novotny, Petra Hruba, Martin Kment, Ludek Voska, Katerina Kabrtova, Antonij Slavcev, Ondrej Viklicky

**Affiliations:** ^1^Department of Nephrology, Transplant Centre, Institute for Clinical and Experimental Medicine, Prague, Czechia; ^2^Institute of Physiology, First Medical Faculty, Charles University, Prague, Czechia; ^3^Transplant Laboratory, Transplant Centre, Institute for Clinical and Experimental Medicine, Prague, Czechia; ^4^Department of Clinical and Transplant Pathology, Institute for Clinical and Experimental Medicine, Prague, Czechia; ^5^Department of Immunogenetics, Institute for Clinical and Experimental Medicine, Prague, Czechia

**Keywords:** antibody-mediated rejection, intimal arteritis, kidney transplantation, vascular rejection, rejection diagnostics

## Abstract

**Background:** The prognostic role of intimal arteritis of kidney allografts in donor-specific antibody negative (DSA–) antibody-mediated rejection (ABMR) remains unclear.

**Methods:** Seventy-two out of 881 patients who had undergone kidney transplantation from 2014 to 2017 exhibited intimal arteritis in biopsies performed during the first 12 months. In 26 DSA negative cases, the intimal arteritis was accompanied by tubulointerstitial inflammation as part of T cell-mediated vascular rejection (TCMRV, *N* = 26); intimal arteritis along with microvascular inflammation occurred in 29 DSA negative (ABMRV/DSA–) and 19 DSA positive cases (ABMRV, DSA+, *N* = 17). In 60 (83%) patients with intimal arteritis, the surveillance biopsies after antirejection therapy were performed. Hundred and two patients with non-vascular ABMR with DSA (ABMR/DSA+, *N* = 55) and without DSA (ABMR/DSA–, *N* = 47) served as controls. Time to transplant glomerulopathy (TG) and graft failure were the study endpoints.

**Results:** Transplant glomerulopathy -free survival at 36 months was 100% in TCMRV, 85% in ABMR/DSA–, 65% in ABMRV/DSA-, 54% in ABMR/DSA+ and 31% in ABMRV/DSA+ (log rank *p* < 0.001). Death-censored graft survival at 36 months was 98% in ABMR/DSA-, 96% in TCMRV, 86% in ABMRV/DSA–, 79% in ABMR/DSA+, and 64% in ABMRV/DSA+ group (log rank *p* = 0.001). In surveillance biopsies, the resolution of rejection was found in 19 (90%) TCMRV, 14 (58%) ABMRV/DSA–, and only 4 (27%) ABMRV/DSA+ patients (*p* = 0.006). In the multivariable model, intimal arteritis as part of ABMR represented a significant risk for TG development (HR 2.1, 95% CI 1.2–3.8; *p* = 0.012) regardless of DSA status but not for graft failure at 36 months.

**Conclusions:** Intimal arteritis as part of ABMR represented a risk for early development of TG regardless of the presence or absence of DSA. Intimal arteritis in DSA positive ABMR represented the high-risk phenotype.

## Introduction

Antibody-mediated rejection (ABMR) represents a major obstacle in achieving long-term graft function and survival (1). Its diagnosis is based on histology and detection of donor-specific antibodies (DSA) (2). Overlapping phenotypes and cases with incomplete manifestation represent a considerable part of diagnoses in clinical practice (3).

Intimal arteritis, i.e., v-lesion defined by Banff histological criteria, is a morphologic feature of vascular rejection. It represents a diagnostic and therapeutic challenge as it is involved in histologic criteria of both T cell-mediated rejection (TCMR) and ABMR (2). Although, previously intimal arteritis was recognized as the rejection phenotype often resistant to steroid treatment (4), more recently, the association of intimal arteritis with DSA was described (5). Lefaucheur et al. revealed a detrimental impact of intimal arteritis associated with DSA on graft prognosis exceeding other rejection phenotypes, and thus any grade of intimal arteritis has been included in the Banff classification as a histologic feature of ABMR (6).

Almost half of the patients with histological features of ABMR in DSA negative patients do not fit the current Banff classification (7). Intimal arteritis is frequently present in both TCMR and ABMR occurring early after kidney transplantation (8). Although a negative prognostic role of DSA in ABMRV has been well-documented (5), the outcome of ABMRV in DSA negative patients remains poorly understood. Therefore, in this study, we compared the outcome of intimal arteritis as part of both T cell-mediated vascular rejection (TCMRV) and antibody-mediated vascular rejection (ABMRV) with ABMR without intimal arteritis in patients with and without DSA in terms of premature graft loss and transplant glomerulopathy (TG) development.

## Materials and Methods

### Study Design and Population

**Figure 1:**
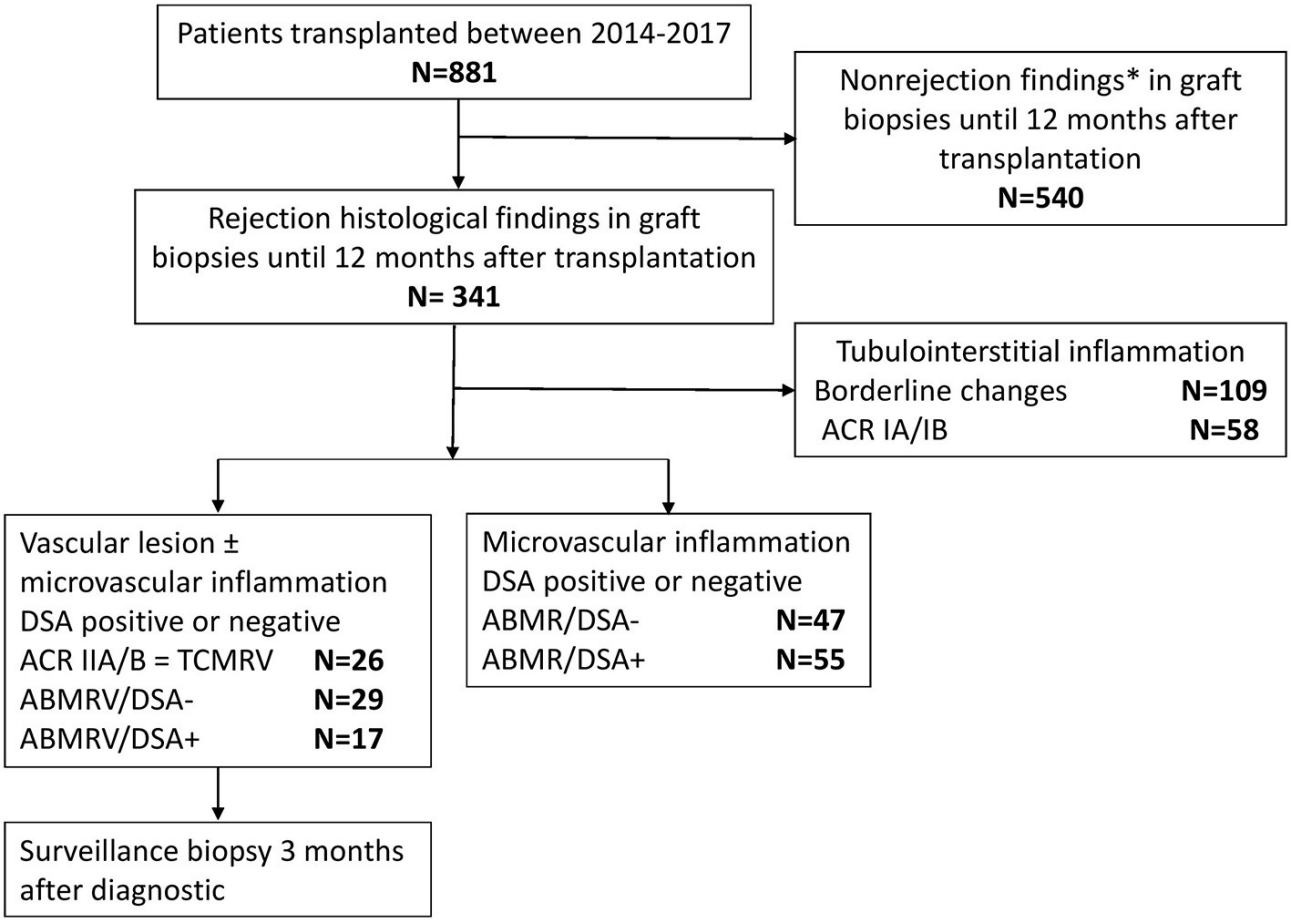
Study flowchart. * Including parainfectious changes, tubulointerstitial inflammation in the presence of BKV, and suspected recurrence of glomerulonephritis.

In this single center retrospective observational cohort study, we evaluated the outcome and clinical relevance of intimal arteritis in the early posttransplant biopsies performed within 12 months from January 2014 to December 2017. For comparison, we retrospectively reviewed medical records of 881 patients who had undergone kidney transplantation at the same time to identify those with histologic features of ABMR in early biopsies and enrolled them for further investigation. Data collection was finalized in January 2021 when all the study subjects reached a 3-year follow-up. Demographics of the study cohort are given in **Table 2** and the enrollment is described in Figure 1. All patients signed the informed consent with case or surveillance biopsies and medical data assessment, and the local Ethical Board approved the study under No.: 15-265191A.

### Histopathology and the Definition of Rejection Phenotype Categories

Kidney allograft biopsies were performed using a percutaneous ultrasound-guided 16G biopsy needle. Diagnoses of acute rejection were established at a median time 70 days after transplantation by for-cause biopsies in 120 (69%) of patients. Fifty-two (31%) rejections were found in protocol biopsies at the third month after transplantation.

Biopsy samples were assessed for Banff scored lesions (2) as glomerulitis (g), peritubular capillaritis (ptc), transplant glomerulopathy (cg), intimal arteritis (v), interstitial inflammation (i), tubulitis (t), mesangial matrix increase (mm), vascular intimal fibrosis (cv), arteriolar hyaline thickening (ah), interstitial fibrosis (ci), or tubular atrophy (ct). The microvascular inflammation (MVI) score was defined as the sum of glomerular (g) and peritubular capillary inflammation (ptc). Immunofluorescence detection of C4d was performed in all cases.

Patients with glomerulitis, peritubular capillaritis, intimal arteritis, and/or TG were divided into five groups (TCMRV *N* = 26, ABMRV/DSA– *N* = 29, ABMRV/DSA+ *N* = 17, ABMR/DSA– *N* = 47, ABMR/DSA+ *N* = 55) according to the histopathological finding and presence or absence of DSA (Table 1).

**Table 1:** Definition of the rejection phenotype groups.

	**g**	**ptc**	**v**	**I**	**t**	**C4d**	**DSA**
TCMRV	0	≥0^*^	≥1	0–3	0–3	0	Neg
ABMRV/DSA–	0-3	≥0^*^	≥1	0–3	0–3	0–3	Neg
ABMRV/DSA+	0–3	≥0^*^	≥1	0–3	0–3	0–3	Pos
ABMR/DSA–	0–3	≥0^*^	0	0–3	0–3	0–3	Neg
ABMR/DSA+	0–3	≥0^*^	0	0–3	0–3	0–3	Pos

* *In presence of tubulointerstitial inflammation ptc alone was not evaluated as ABMR criterion*.

T cell-mediated vascular rejection was characterized by intimal arteritis and tubulointerstitial inflammation (TI) in the absence of glomerulitis of rejection origin, C4d, and DSA. ABMRV included features of MVI and C4d (positive or negative) in addition to v-lesion. Patients were further divided according to their DSA status as ABMRV/DSA– when DSA were negative and ABMRV/DSA+ when DSA were positive. ABMR, defined by the presence of MVI and C4d (positive or negative), were further divided according to DSA status as described above (ABMR/DSA–, ABMR/DSA+). Four phenotypes fulfilling criterion 1 (histologic evidence of acute tissue injury) and 2 (evidence of recent antibody interaction with endothelium) of Banff ABMR definition were called histologic ABMR groups.

### Anti-HLA Antibody Testing

Identification of circulating donor-specific anti-HLA antibodies (DSA) was performed by Luminex bead-based assay (One Lambda Inc., Canoga Park, CA, USA). Class I A, B, and class II DR antibodies were evaluated for specificity in all recipients before transplantation. Class II DP and DQ antibodies were evaluated for specificity after transplantation because donors' HLA DP or DQ typing was not available at the time of transplantation. DSA positivity at the time of transplantation was revealed in 14 (82%) of the patients with ABMRV/DSA+ and 47 (86%) of them with ABMR/DSA+. In the rest of the patients from these phenotype categories, DSA was detected at the time of diagnostic biopsy.

### Immunosuppressive Treatment

Patients received maintenance immunosuppression based on tacrolimus, mycophenolate mofetil, and corticosteroids. All patients received induction treatment. Patients at low risk (*N* = 73) received basiliximab whereas those at high risks, such as retransplants and patients with anti-HLA antibodies or DSA, received rabbit anti-thymocyte globulin (rATG) (*N* = 49). Patients with DSA positivity (<5,000 MFI) at transplant had undergone plasma exchange (PE) prior to transplantation and intravenous immunoglobulin (IVIG) as additional treatment (*N* = 52). The later described multivariable Cox regression model was adjusted based on induction immunosuppression to eliminate confounding variables.

Patients with rejection were treated by steroid pulses [18 (69%) TCMVR; 8 (28%) ABMRV/DSA–; 1 (6%) ABMRV/DSA+; 37 (79%) ABMR/DSA-; 17 (31%) ABMR/DSA+] or rATG [7 (27%) TCMRV; 15 (52%) ABMRV/DSA–; 3 (18%) ABMRV/DSA+; 2 (4%) ABMR/DSA–, none of ABMR/DSA+] and/or plasmapheresis/IVIG [1 (4%) TCMRV; 6 (21%) ABMRV/DSA-; 13 (77%) ABMRV/DSA+; 5 (11%) ABMR/DSA–; 33 (60%) ABMR/DSA+]. Altogether, eight patients were not treated mostly due to concomitant infectious complications.

### Surveillance and Subsequent Biopsies

Altogether, 124 patients from the whole cohort underwent at least one biopsy after the diagnostic examination. All patients who experienced intimal arteritis in early biopsies were eligible for surveillance biopsy at 3 months after the first biopsy. After obtaining written informed consent, 60 out of the 72 patients (83%) who experienced intimal arteritis underwent the surveillance biopsy. Patients from ABMR/DSA– and ABMR/DSA+ groups underwent subsequent biopsies either at the time of center protocol biopsy at the third month or when clinically indicated due to graft function worsening or proteinuria.

### Statistical Analyses

Continuous variables were expressed as median and range. Categorical variables were expressed as *n* and percentage of the total. Categorical Banff histologic scores were expressed as count per category. The Chi-square, ANOVA, Kruskal–Wallis, and Wilcoxon tests were used for hypothesis testing when appropriate. *p* < 0.05 were considered statistically significant. Survival analyses were performed with the Kaplan–Meier method using the log-rank test. To identify factors associated with death-censored graft failure and the development of TG, univariable Cox regression was created. For the multivariable model, all variables with *p* < 0.1 were selected, and only variables without missing data were included. Data analyses were performed using IBM SPSS 22 (SPSS, Inc. Chicago, IL), R Studio 4.0.3. (2020-10-10), and GraphPad Prism5 (Graph Pad, San Diego, CA).

## Results

### Demographics and Clinical Characteristics

In 174 patients, five different rejection phenotypes were identified: 26 TCMRV, 29 ABMRV/DSA–, 17 ABMRV/DSA+, 47 ABMR/DSA–, and 55 ABMR/DSA+ (Table 1). Diabetes, ischemic nephropathy, and glomerulonephritis were the most common original diseases. DSA– phenotypes were more common after the first transplantation. DSA positive phenotypes were more frequent in retransplantation. Peak PRA was significantly higher in DSA positive groups. Cohort demographics are given in Table 2.

**Table 2:** Overview of the baseline characteristics of all rejection phenotype groups.

	**Total**	**TCMRV**	**ABMRV/DSA–**	**ABMRV/DSA+**	**ABMR/DSA-**	**ABMR/DSA+**	***P*-value**
	***N* = 174**	***N* = 26**	***N* = 29**	***N* = 17**	***N* = 47**	***N* = 55**	
**Age, yr**	53 (21–81)	54 (23–70)	56 (23–81)	49 (25–78)	56 (21–71)	48 (23–78)	0.121
**Gender (female)**	62 (36)	8 (31)	11 (38)	5 (29)	15 (32)	23 (42)	0.768
**Dialysis vintage, m**	26 (0–263)	20 (0–263)	25 (0–118)	32 (0–131)	21 (0–128)	38 (0–139)	0.046
**PRA max, %**	7 (0–100)	2 (0–69)	2 (0–22)	34 (0–100)	3 (0–98)	32 (0–100)	**<0.001**
**HLA mismatch**	3 (0–6)	4 (1–6)	3 (0–6)	4 (2–6)	3 (0–6)	3 (1–6)	0.14
**1st transplantation**	123 (71)	24 (92)	27 (93)	8 (47)	45 (96)	19 (35)	**<0.001**
**DSA at Tx**, ***n*** **(%)**	61 (35)	0	0	14 (82)	0	47 (86)	0.756^*^
DSA class I, *n* (%)	23 (13)	0	0	3 (18)	0	20 (36)	0.148^*^
DSA class II, *n* (%)	28 (16)	0	0	8 (46)	0	20 (36)	0.720^*^
DSA both classes, *n* (%)	21 (12)	0	0	6 (36)	0	15 (28)	0.525^*^
FACS T at biopsy	104/19 (18)	14/2 (14)	26/3 (12)	14/3 (21)	18/0	32/10 (31)	**<0.001**
FACS B at biopsy	104/31 (29)	14/1 (7)	26/0	14/8 (57)	18/1 (5)	32/21 (65)	**<0.001**
**Cold ischemia, h**	14 (0.3–24)	13 (0.3–21)	13 (0.3–20)	16 (0.5–23)	14 (0.5–23)	15 (0.6–24)	0.26
**Deceased donor**, ***n*** **(%)**	146 (84)	17 (65)	23 (79)	15 (88)	39 (83)	52 (95)	0.018
**CKD diagnosis**							0.039
Diabetes	23 (13)	3 (12)	6 (21)	2 (12)	11 (23)	1 (2)	
Vascular + TIN	41 (24)	10 (39)	8 (28)	5 (29)	6 (13)	12 (22)	
Glomerulonephritis	50 (29)	4 (15)	5 (17)	5 (29)	14 (30)	22 (40)	
Hereditary nephropathy	35 (20)	6 (23)	5 (17)	1 (6)	8 (17)	15 (27)	
Other	25 (14)	3 (12)	5 (17)	4 (24)	8 (17)	5 (9)	
**Induction therapy**							**<0.001**
Basiliximab	73 (42)	17 (65)	22 (76)	2 (12)	28 (60)	4 (7)	
rATG	49 (28)	8 (31)	7 (24)	7 (41)	15 (32)	12 (22)	
rATG, PE, IVIG	52 (30)	1 (4)	0	8 (47)	4 (8)	39 (71)	
**Maintenance IS**							
**Tac/MMf/steroids**	163 (94)	25 (96)	25 (86)	15 (88)	44 (94)	54 (98)	0.058
**Time of diagnosis, d**	70 (2–357)	74 (3–204)	30 (5–357)	24 (7–351)	80 (2–301)	61 (5–323)	0.976
**For–cause biopsy**	120 (69)	17 (65)	25 (86)	16 (94)	26 (55)	36 (66)	**0.009**
**Treatment of rejection**							**<0.001**
Methylprednisolone	81	18 (69)	8 (28)	1 (6)	37 (79)	17 (31)	
rATG	27	7 (27)	15 (52)	3 (18)	2 (4)	0	
PE, IVIG	58	1 (4)	6 (21)	13 (76)	5 (11)	33 (60)	
None	8	0	0	0	3 (6)	5 (9)	

* *Comparing ABMR/DSA+ and ABMRV/DSA+ rejection phenotypes*.

### Surveillance Biopsies

**Figure 2:**
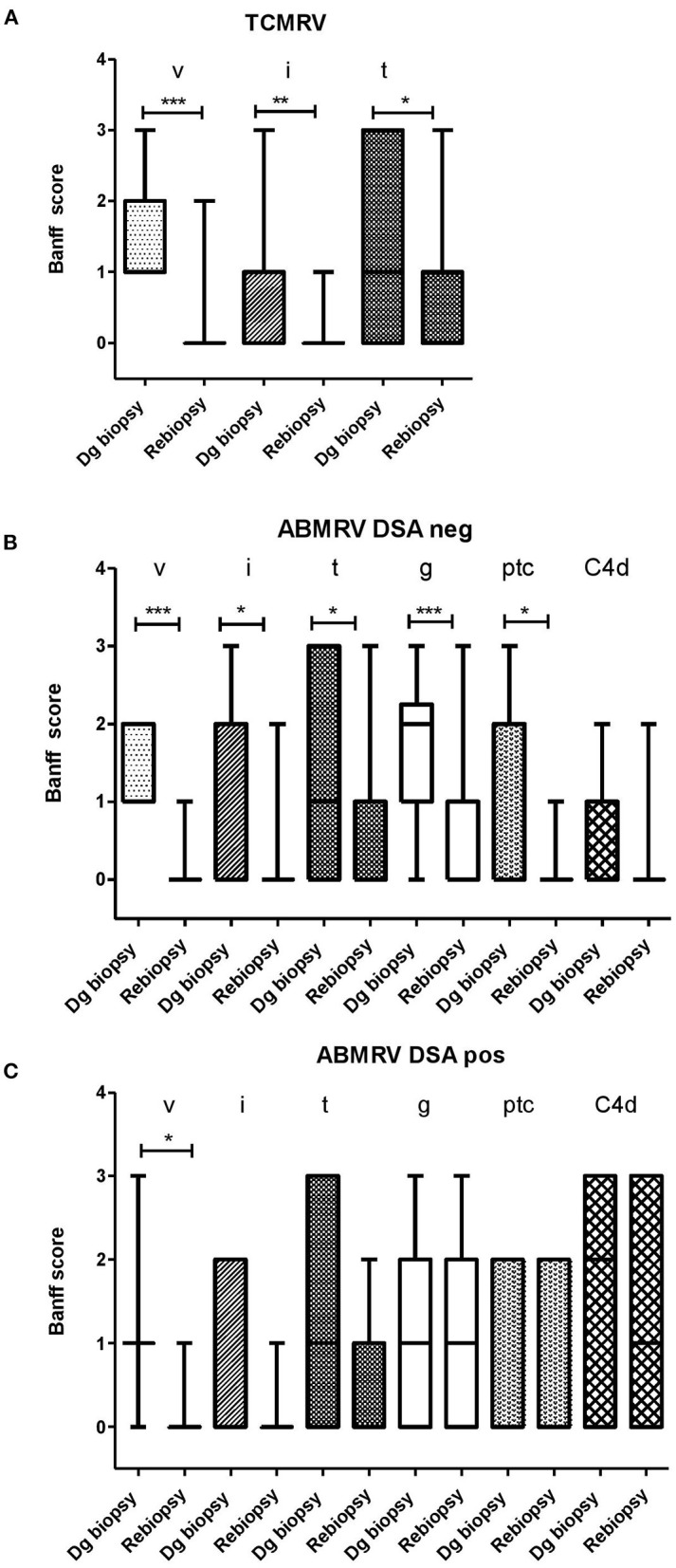
The development of Banff score between diagnostic (dg biopsy) and rebiopsies in a subgroup of patients with consented rebiopsies. **(A)** TCMRV (*n* = 21) **(B)** ABMRV DSA– (*n* = 22) and **(C)** ABMRV DSA+ (*n* = 15). Differences were calculated by Wilcoxon matched-paired signed rank test. *p < 0.05, **p < 0.01, and ***p < 0.001.

Surveillance biopsies at 3 months after vascular rejection diagnosis were performed in 21 out of 26 patients (81%) with TCMRV, 24 out of 29 (83%) patients with DSA negative ABMRV, 15 out of 17 (88%) patients with DSA positive ABMRV. At case biopsy, DSA negative patients with ABMRV did not differ from DSA positive ABMRV patients in any of the histologic scores, indicating acute inflammation (g, ptc, i, t, v). The only significant difference was the intensity of C4d (*p* = 0.003). Surveillance biopsies revealed the resolution of rejection in 19 (90%) TCMRV cases, 14 (58%) ABMRV/DSA- cases, and only four (27%) ABMRV/DSA+ cases (*p* = 0.006). When comparing case and surveillance biopsies, patients with TCMRV had significantly improved i, t, and v scores, and DSA– patients with ABMRV had significantly improved in g, ptc, i, t, and v scores; however, in DSA+ ABMRV cases, only the v-score had improved (Figure 2, Table 3).

**Table 3:** Comparison of Banff scores between diagnostic and surveillance biopsies for patients with intimal arteritis.

	**TCMRV**			**ABMRV/DSA–**			**ABMRV/DSA+**		
	**Diagnostic biopsy**	**Rebiopsy**	***P*–value**	**Diagnostic biopsy**	**Rebiopsy**	***P*–value**	**Diagnostic biopsy**	**Rebiopsy**	***P*-value**
N	26	21		29	23		17	15	
g	25/1^*^/0/0	18/0/1/0	0.655	7/7/9/6	15/5/1/1	**0.001**	6/7/3/1	7/1/5/2	0.566
ptc	24/1/0/0	19/0/0/0	1.000	14/3/10/2	20/2/0/0	**0.015**	11/1/5/0	11/0/4/0	0.705
i	12/9/3/2	14/4/0/0	**0.005**	14/3/8/4	19/2/1/0	**0.021**	10/3/4/0	13/2/0/0	**0.045**
t	11/4/4/7	11/5/1/1	**0.010**	6/8/6/9	16/2/1/3	**0.018**	9/3/1/4	10/4/1/0	0.075
ti	11/9/4/2	13/4/1/0	**0.020**	3/8/13/5	14/3/4/1	**0.028**	6/4/3/4	5/6/1/3	0.928
v	0/19/6/1	18/0/1/0	**<0.001**	0/20/9/0	20/2/0/0	**<0.001**	0/14/2/1	12/3/0/0	**0.003**
ci	7/19/0/0	3/15/0/0	0.705	5/20/3/0	5/11/6/0	0.405	6/11/0/0	3/11/1/0	0.102
ct	4/21/1/0	0/19/0/0	1.000	1/24/3/0	3/13/6/0	0.480	0/17/0/0	0/14/1/0	0.317
cg	25/1^*^/0/0	19/0/0/0	0.317	26/1/2/0	16/4/2/0	0.180	15/2/0/0	8/4/2/1	**0.015**
cv	3/16/5/2	2/12/3/3	1.000	5/12/9/2	1/15/3/2	0.439	3/6/6/2	4/5/5/1	0.565
ah	1/21/4/0	3/10/5/1	0.739	3/17/8/1	3/14/5/0	0.527	4/6/7/0	1/9/5/0	0.608
C4d	20/5/0/1^#^	16/4/0/0	0.366	20/6/2/1	18/3/1/0	0.317	5/2/2/8	4/4/3/4	0.336
IF/TA	7/17/1/0	3/15/0/0	0.527	5/20/3/0	5/11/6/0	0.405	6/10/1/0	3/11/1/0	0.257

*Differences were calculated by the Wilcoxon matched–paired signed–rank test*.

* *Suspicious for recurrence of the original disease*.

# *ABO incompatible transplantation, assumption of accommodation*.

*Bold P-values represent statistic significance below 0.05*.

### Development of TG

**Figure 3:**
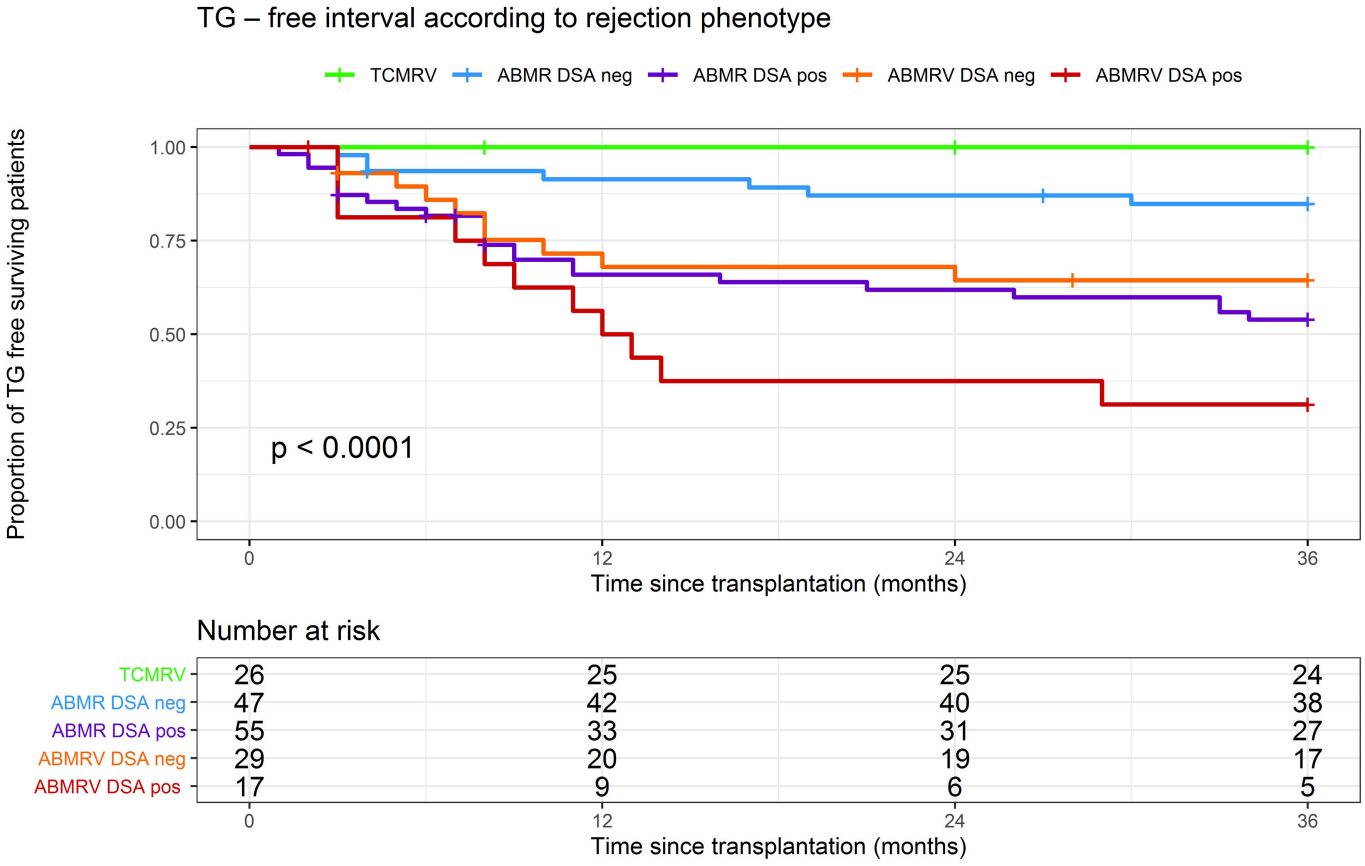
Kaplan–Meier analysis of TG-free interval according to rejection phenotype.

Significant differences between ABMR rejection phenotypes were found when we studied the development of the TG. For cause and surveillance, biopsies at the 3-year follow-up were included in the analysis. Estimate of the cumulative proportion of patients without TG (TG-free survival) at 36 months was 100% in TCMRV, 85% in ABMR/DSA-, 65% in ABMRV/DSA–, 54% in ABMR/DSA+, and 31% ABMRV/DSA+ (log rank *p* < 0.001). Pairwise comparisons showed a significantly longer time to TG in the TCMRV group than in other groups (log rank *p* < 0.05). Interestingly, DSA– ABMRV patients had a significantly shorter time to TG development than DSA– ABMR patients (mean for survival time without TG was 26 and 32 months, respectively) (log rank *p* = 0.035) (Figure 3).

Cox regression models were performed to identify risk factors for the development of TG in patients with histologic ABMR. Risk factors identified in the univariable analysis were included in the multivariable model (retransplantation status, DSA positivity, rATG therapy, rATG + PE/IVIG therapy, intimal arteritis at case biopsy, and C4d positivity). Multivariable model adjusted for the variables mentioned above found intimal arteritis to be associated with TG development (HR 2.1, *p* = 0.01) (Table 4).

**Table 4:** Cox regression assessing risk factors of development of transplant glomerulopathy (No. of events is 52) in histologic ABMR rejection categories (ABMR/DSA–, ABMR/DSA+, ABMRV/DSA–, ABMRV/DSA+).

**Variable**	**Univariable analysis**	***p*–value**	**Variable**	**Univariable analysis**	***p*–value**
	**HR (95% CI)**			**HR (95% CI)**	
**ABMRV/DSA–**	1.0 (0.5–2.0)	0.972	**Basiliximab**	0.4 (0.2–0.7)	**0.002**
**ABMRV/DSA+**	2.7 (1.4–5.2)	**0.004**	**rATG±PE/IVIG**	2.8 (1.4–5.5)	**0.002**
**ABMR/DSA–**	0.3 (0.1–0.6)	**0.001**	**Diagnostic biopsy**		
**ABMR/DSA+**	1.6 (1.0–2.8)	0.076	g>0	1.0 (0.5–2.1)	0.937
**Donor (living)**	0.5 (0.2–1.4)	0.179	ptc>0	1.4 (0.8–2.4)	0.256
**PRA>20%**	1.2 (0.7–2.1)	0.508	i>0	0.8 (0.4–1.5)	0.445
**DSA positivity**	2.7 (1.5–4.8)	**0.001**	t>0	1.3 (0.7–2.3)	0.351
**HLA mm>3**	1.4 (0.8–2.4)	0.244	ti>1	0.9 (0.5–1.6)	0.642
**HD vintage>** **3y**	0.9 (0.5–1.5)	0.609	v>0	1.7 (1.0–2.9)	0.064
**CKD diagnosis**			ci>1	1.2 (0.4–3.9)	0.727
Diabetes	1.2 (0.6–2.6)	0.588	ct>1	1.2 (0.4–3.9)	0.727
Vascular	1.1 (0.6–2.2)	0.692	cg>0	NA	NA
Glomerulonefritis	0.7 (0.4–1.3)	0.277	cv>1	1.0 (0.6–1.7)	0.947
Hereditary	1.1 (0.6–2.1)	0.826	ah>1	1.1 (0.6–1.9)	0.724
Other	1.1 (0.5–2.3)	0.822	IF/TA>1	1.6 (0.6–4.3)	0.388
**Retransplantation**	1.8 (1.0–3.1)	**0.040**	C4d>1	1.7 (1.0–3.0)	0.068
	**Multivariable analysis**			**Multivariable**	
	**HR (95% CI)**			**HR (95% CI)**	
**Retransplantation**	0.8 (0.4–1.5)	0.395	**rATG** **±** **PE/IVIG**	2.4 (1.0–5.6)	0.055
**DSA positivity**	2.1 (1.0–4.7)	0.064	**v>0**	2,1 (1.2–3.8)	**0.012**
			**C4d>1**	1.2 (0.6–2.3)	0.614

*Bold P-values represent statistic significance below 0.05*.

### Kidney Graft Survival

**Figure 4:**
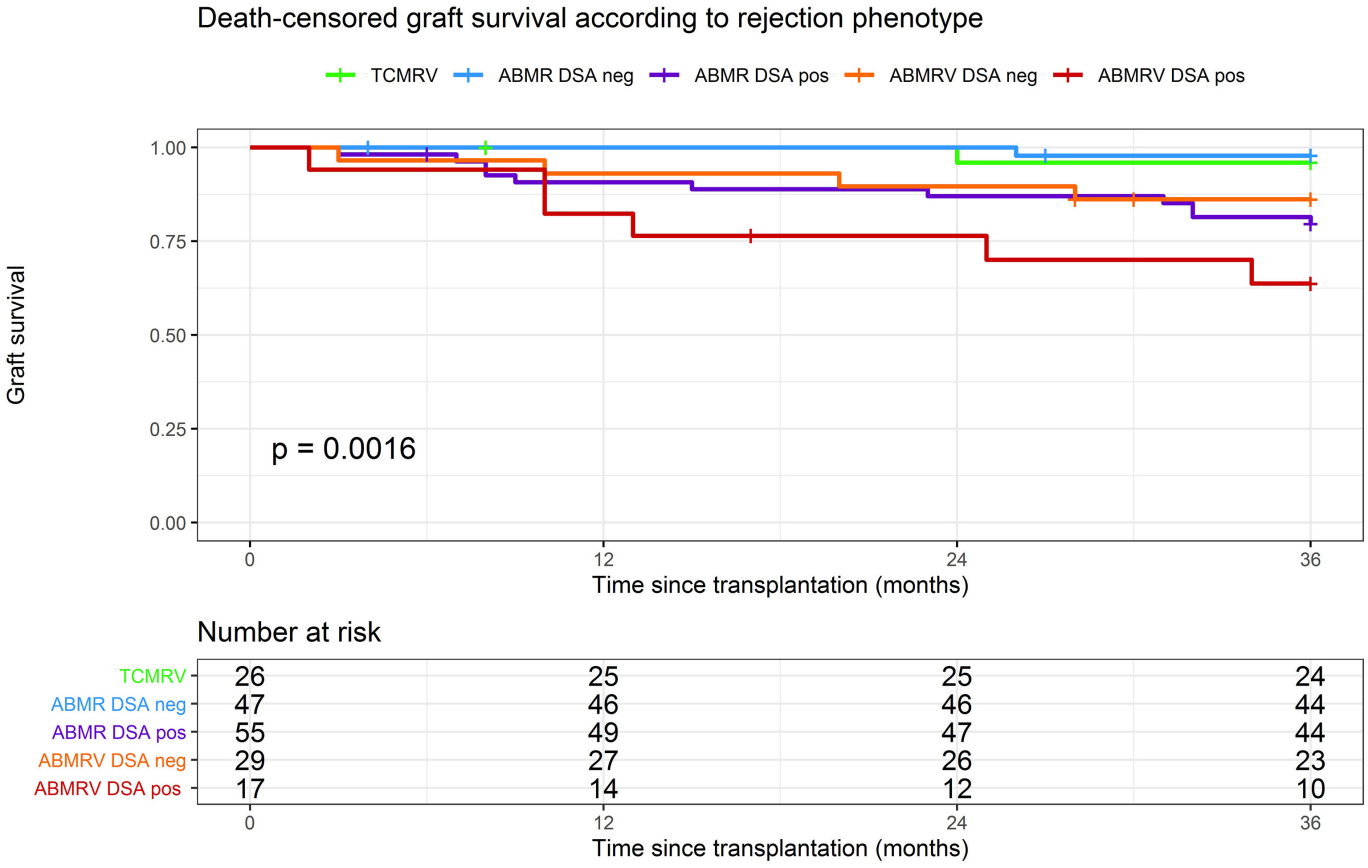
Death-censored graft survival according to rejection phenotype.

The graft survival was significantly impaired in DSA+ patients with intimal arteritis as a part of ABMR compared with others (Figure 4). Death-censored kidney graft survival at 36 months was 96% in TCMRV, 98% in ABMR/DSA–, 86% in ABMRV/DSA–, 79% in ABMR/DSA+, and 64% in the ABMRV/DSA+ group (log rank *p* = 0.001). Interestingly, DSA– patients with intimal arteritis as part of ABMR experienced similar graft survival as DSA+ patients with ABMR (*p* = 0.507).

Cox regression assessing risk factors for graft failure in ABMR revealed PRA > 20% (HR 4.4, *p* = 0.001), DSA positivity (HR 3.9, *p* = 0.007), retransplantation (HR 4.0, *p* = 0.002), induction with ATG and ATG with additional PE, IVIG (HR 4.1, *p* = 0.021), and C4d positivity (HR 2.5, *p* = 0.041) in the univariable analysis (Table 5). None of these variables were found to be significant in the multivariable model.

**Table 5:** Cox regression assessing risk factors of graft failure (No. of events 23) in histologic ABMR rejection categories (ABMR/DSA–, ABMR/DSA+, ABMRV/DSA–, and ABMRV/DSA+).

**Variable**	**Univariable analysis**	***p*–value**	**Variable**	**Univariable analysis**	***p*–value**
	**HR (95% CI)**			**HR (95% CI)**	
**ABMRV/DSA–**	0.9 (0.3–2.7)	0.864	**Basiliximab**	0.2 (0.1–0.8)	**0.021**
**ABMRV/DSA+**	3.3 (1.3–8.6)	**0.011**	**rATG±PE/IVIG**	4.1 (1.2–14.1)	**0.021**
**ABMR/DSA–**	0.1 (0.0–0.7)	**0.020**	**Diagnostic biopsy**		
**ABMR/DSA+**	1.7 (0.8–4.0)	0.192	g>0	0.6 (0.2–1.5)	0.294
**Donor (living)**	0.3 (0.0–2.2)	0.239	ptc>0	1.6 (0.7–3.7)	0.295
**PRA>20%**	4.4 (1.8–10.8)	**0.001**	i>0	1.5 (0.6–3.4)	0.400
**DSA positivity**	3.9 (1.4–10.6)	**0.007**	t>0	1.3 (0.6–3.0)	0.574
**HLA mm>3**	1.5 (0.7–3.6)	0.334	ti>1	1.3 (0.6–3.2)	0.527
**HD vintage>** **3y**	1.1 (0.5–2.5)	0.871	v>0	1.9 (0.9–4.6)	0.112
**CKD diagnosis**			ci>1	0.9 (0.1–6.7)	0.918
Diabetes	0.04 (0.0–7.0)	0.221	ct>1	0.9 (0.1–6.7)	0.918
Vascular	1.8 (0.7–4.5)	0.185	cg>0	0.04 (0.0–20.0)	0.314
Glomerulonefritis	1.3 (0.5–3.1)	0.570	cv>1	1.9 (0.8–4.3)	0.145
Hereditary	0.9 (0.3–2.6)	0.792	ah>1	1.5 (0.7–3.6)	0.312
Other	1.0 (0.3–3.2)	0.943	IF/TA>1	1.7 (0.4–7.6)	0.441
**Retransplantation**	4.0 (1.7–9.6)	**0.002**	C4d>1	2.5 (1.0–6.3)	**0.041**

*Bold P-values represent statistic significance below 0.05*.

## Discussion

Intimal arteritis, a diagnostic feature of vascular rejection, is a frequent histological finding in kidney allografts (5, 8, 9). In this study, we found that intimal arteritis as part of histologic ABMR represents a risk for early development of TG regardless of the presence or absence of donor-specific anti-HLA antibodies. Therefore, a poor outcome of this phenotype is anticipated. On the other hand, we found that intimal arteritis as part of TCMR has a favorable kidney graft outcome when standard antirejection therapy is applied. Similar to our data, molecular assessment of kidney allografts revealed the early occurrence of isolated intimal arteritis of benign origin after transplantation (10–12).

Previously, vascular rejection, i.e., intimal arteritis, was characterized by a high rate of steroid-resistance and poor kidney allograft outcomes (4, 13). Just recently, the intimal arteritis was shown to be associated with DSA with the detrimental impact of this rejection phenotype on graft survival and was accepted as a diagnostic criterion for ABMR (5, 6).

Interestingly, in DSA– patients with intimal arteritis as part of histologic ABMR, the occurrence of TG was higher than in DSA– patients without intimal arteritis despite histologic ABMR. TG is a frequent morphological finding in chronic antibody mediated rejection, and thus, those patients are at the highest risk for premature kidney allograft loss (14).

In our study, the outcome of DSA negative patients with intimal arteritis along with MVI was similar to DSA+ patients with MVI, but without intimal arteritis. One of the possible explanations for this phenomenon is the hypothetical presence of non-HLA antibodies. The association of both MVI and intimal arteritis with anti-angiotensin II type 1 receptor antibodies has been discussed for more than a decade (15, 16). Besides anti-angiotensin II type 1 receptor antibodies, Delville et al. described the association of MVI and intimal arteritis with broader autoimmune reactivity measured *in vitro* by renal microvascular endothelial cells crossmatch assay (17). Very recently, NK cells were found to trigger MVI when a mismatch between donor HLA I and recipient inhibitory killer cell immunoglobulin-like receptor was present (18). This missing self- hypothesis may explain poor outcomes of DSA negative patients with microvascular inflammation. Whether such mechanisms are involved in intimal arteritis as well remains unclear.

Intimal arteritis as part of DSA– ABMR has not been entirely studied. A higher prevalence of AT1R positivity was found along with intimal arteritis. DSA– patients with AT1R positivity lost their grafts prematurely (19). On the other hand, a good prognosis of DSA negative patients with antibody mediated rejection histology was found (7). Graft survival of DSA negative patients with intimal arteritis was not specifically addressed in either of these studies.

There is a lack of studies dealing with graft outcomes in different phenotypes of vascular rejection, especially DSA– histologic ABMR with intimal arteritis, since the major Banff classification update in 2013 (6). Rabant et al. analyzed the outcome of patients with early isolated v-lesions and found better outcomes than in other phenotypes (10). Shimizu et al. observed similar outcomes in patients with intimal arteritis as part of both TCMR and ABMR; however, the study cohort was limited to 31 patients (20). Wu et al. compared the outcome of patients with intimal arteritis, classified both according to the grade of intimal arteritis and Banff classification and found that the grade plays a more important role than the Banff category (21). Salazar et al. reported nine out of 10 graft failures in patients with v-lesions (11). Patient numbers in all the mentioned studies above are small, which limits their conclusions. It is important to note that the repeated updates of Banff classifications on ABMR make the interpretation of former studies problematic (22).

Limitations of our study were a short follow-up period which confined the number of study endpoints, and above all, we were not able to demonstrate the significant impact of vascular rejection in the absence of DSA on graft survival. The incomplete number of surveillance biopsies in the control groups was limiting direct comparison of all rejection phenotypes. The study did not involve the evaluation of non-HLA antibodies or transcriptomic data.

In conclusion, intimal arteritis along with MVI was found to represent a risk for the early development of TG regardless of the presence or absence of donor-specific anti-HLA antibodies. Therefore, it is likely that this phenotype reflects the presence of harmful endothelial injury of different origin than humoral alloimmunity.

## Data Availability Statement

The data analyzed in this study is subject to the following licenses/restrictions: Data are stored as medical records of Institute for Clinical and Experimental Medicine, Prague, Czechia. Requests to access these datasets should be directed to Department of Nephrology, Institute for Clinical and Experimental Medicine.

## Ethics Statement

The studies involving human participants were reviewed and approved by Ethical Board of Institute for Clinical and Experimental Medicine approved the study under No.: 15-265191A. The patients/participants provided their written informed consent to participate in this study.

## Author Contributions

MN: data gathering and processing and manuscript writing. PH: serum and sample storage and assessment and data processing. MK and LV: assessment of histological slides. KK and AS: HLA typing and anti-HLA antibodies evaluation. OV: manuscript writing and supervising. All authors contributed to the article and approved the submitted version.

## Funding

This study was supported by the Ministry of Health of the Czech Republic MZO 00023001 and by the Ministry of Health of the Czech Republic under grants NV19-06-00031 and NU21-06-00021.

## Conflict of Interest

The authors declare that the research was conducted in the absence of any commercial or financial relationships that could be construed as a potential conflict of interest.

## Publisher's Note

All claims expressed in this article are solely those of the authors and do not necessarily represent those of their affiliated organizations, or those of the publisher, the editors and the reviewers. Any product that may be evaluated in this article, or claim that may be made by its manufacturer, is not guaranteed or endorsed by the publisher.
